# Bite force in the horned frog (*Ceratophrys cranwelli*) with implications for extinct giant frogs

**DOI:** 10.1038/s41598-017-11968-6

**Published:** 2017-09-20

**Authors:** A. Kristopher Lappin, Sean C. Wilcox, David J. Moriarty, Stephanie A. R. Stoeppler, Susan E. Evans, Marc E. H. Jones

**Affiliations:** 1 Biological Sciences Department, California State Polytechnic University, Pomona, CA 91768 USA; 2 Department of Evolution, Ecology, and Organismal Biology, University of California, Riverside, CA 92521 USA; 3 Department of Cell and Developmental Biology, University College London, London, United Kingdom; 4 School of Biological Sciences, The University of Adelaide, South Australia, 5005 Australia; 5 South Australian Museum, Adelaide, South Australia, 5000 Australia

## Abstract

Of the nearly 6,800 extant frog species, most have weak jaws that play only a minor role in prey capture. South American horned frogs (*Ceratophrys*) are a notable exception. Aggressive and able to consume vertebrates their own size, these “hopping heads” use a vice-like grip of their jaws to restrain and immobilize prey. Using a longitudinal experimental design, we quantified the ontogenetic profile of bite-force performance in post-metamorphic *Ceratophrys cranwelli*. Regression slopes indicate positive allometric scaling of bite force with reference to head and body size, results that concur with scaling patterns across a diversity of taxa, including fish and amniotes (lizards, tuatara, turtles, crocodylians, rodents). Our recovered scaling relationship suggests that exceptionally large individuals of a congener (*C. aurita*) and extinct giant frogs (*Beelzebufo ampinga*, Late Cretaceous of Madagascar) probably could bite with forces of 500 to 2200 N, comparable to medium to large-sized mammalian carnivores.

## Introduction

The evolution of jaws and their use in prey capture has played a key role in the radiation and evolutionary success of vertebrates^1–5^. However, in most non-larval frogs and salamanders, derived tongue projection mechanisms serve as the primary means of capturing prey, and the jaws tend to be weak and play an ancillary role in predation^6,7^. Horned frogs (*Ceratophrys*) are a notable exception.

Although *Ceratophrys* frogs possess large and highly adhesive tongues^8^, they also have strong jaws and, quite atypical of frogs, readily bite. The strength of their bite is reflected by their extremely wide and short heads, which contain large jaw-adductor muscles and provide a high mechanical advantage, even at the tips of the jaws. Using their disproportionately large head and the forceful bite it affords, *Ceratophrys* frogs are capable of capturing and subduing prey that can be large, strong, and/or potentially dangerous (e.g., frogs, lizards, snakes, birds, rodents; ref.^9^).

*In vivo* bite-force performance has been studied in a diversity of vertebrates^10^. Interspecific and intraspecific studies of bite force, including some analyses of ontogenetic variation, have been conducted in various amniotes and some fish, with the results consistently indicating a pattern of positive allometric scaling of bite force with respect to body size and cranial dimensions^11–24^ (Table 1). An understanding of the ontogenetic scaling of biomechanical performance provides a rare opportunity to gain insight into the ecology and behavior of extinct taxa, including those beyond the size range of extant analogues^22,24^.

**Table 1:** Published studies of intraspecific scaling of *in vivo* voluntary bite force.

Taxon^1^	Experimental Design	Morphological Variable^2^	Slope	Citation
fish	latitudinal	total length	**2.30**	22. Grubich *et al*. 2012
lizards (2)	latitudinal	snout-vent length	**4.60, 3.83**	13. Meyers *et al*. 2002
lizards (2)	latitudinal	head width	**3.24, 2.89**	17. Herrel & O’Reilly 2006
tuatara	latitudinal	snout-vent length	**2.72/3.49** ^3^	37. Jones & Lappin 2009
alligators	latitudinal	jaw length	**2.57**	14. Erickson *et al*. 2003
turtles (3)	latitudinal	head width	2.40^4^, 2.10^4^, **2.63**	17. Herrel & O’Reilly 2006
turtles	latitudinal	head width	**2.44**	21. Pfaller *et al*. 2010
rodents	latitudinal	mandibular width	**2.88**	38. Becerra *et al*. 2011
rodents	latitudinal	mandibular width	2.05^5^	39. Becerra *et al*. 2013
frogs	longitudinal	head width	**3.3269**	this study

^1^Number of species given in parentheses if more than one was examined in the study.^ 2^If reported, the slope of bite force on head width is given. If not, then the slope of bite force on another morphometric is given. ^3^Estimated at most anterior and most posterior teeth. ^4^Slope of bite force on other head dimensions showed positive allometry. ^5^Slope of bite force on body mass showed positive allometry. Cases of significant positive allometry in bold.

Here, we present an analysis of the ontogenetic scaling of *in vivo* bite force in *Ceratophrys cranwelli* ^25^ (Cranwell’s Horned Frog^26^). Using a longitudinal experimental design, we measured bite-force performance in a sample of eight post-metamorphic individuals to test the hypothesis that, typical of fish and amniotes examined to date, this non-amniote tetrapod exhibits a positive allometric increase in bite force during ontogeny. We then use our data to extrapolate the maximum bite-force performance of two other taxa, an exceptionally large museum specimen of an extant congener (*C. aurita*), as well as a giant ceratophryid*-*like frog from the Late Cretaceous of Madagascar (*Beelzebufo* ^27,28^) that lived contemporaneously with small crocodylians and non-avian dinosaurs that were potential prey. In light of our results, we discuss the role of a forceful bite and associated specializations in shaping the unusual dietary behavioral ecology of these megalophagous predators.

## Results

### Scaling of bite-force performance in *Ceratophrys cranwelli*

**Figure 1:**
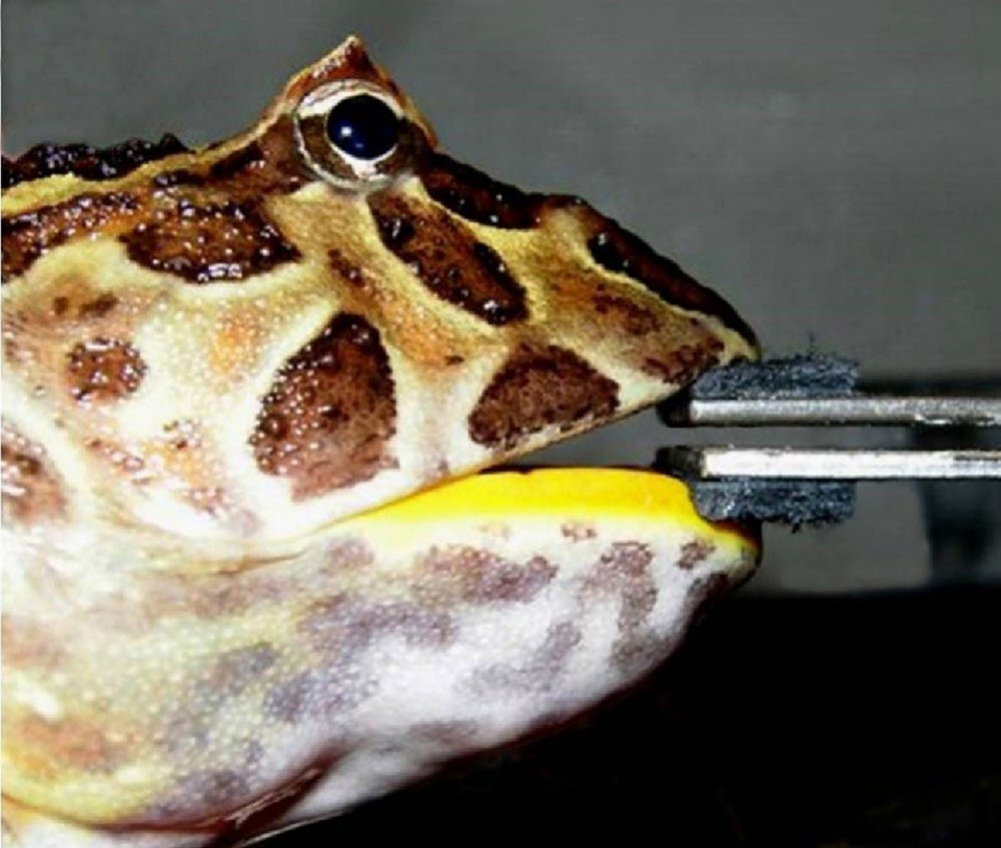
An individual *Ceratophrys cranwelli* biting a force transducer. Leather strips glued to ends of bite bars provide a natural surface that encourages high-effort biting and avoids damage to teeth and bones. The strips also indicate a bite point for standardization of bite-force performance (see Methods).

Our sample of eight frogs spanned a nearly 17-fold range in body mass (8.9–147.8 g) and a 1.9 to 2.4-fold range in linear morphometrics of the body (length: 39.8–95.6 mm) and head (length: 14.7–31.7 mm; width: 22.4–46.1 mm; depth: 10.8–20.6 mm; Supplementary Table S1). Direct measurement of bite force (Fig. 1) produced voluntary performance that varied 12-fold (2.7–32.9 N).

**Figure 2:**
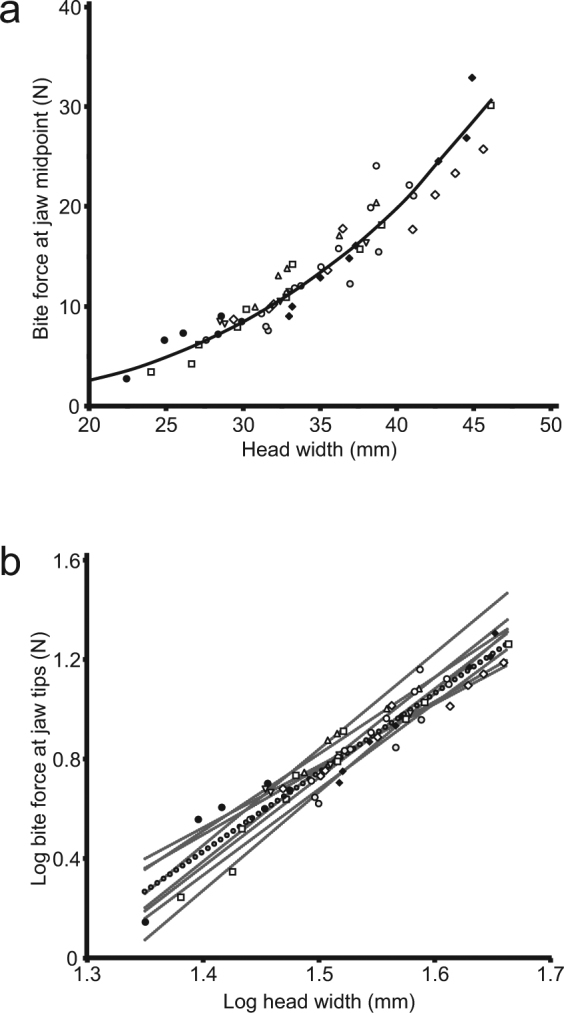
Head width vs. bite force for specimens of *Ceratophrys cranwelli*. (**a**) Plot of bite force on head width in which each point represents maximum bite force of three trials (standardized here for bite position at posterior teeth, see Methods) measured during an experimental session with each specimen represented by a different symbol (*n* = 8). The line represents a power function fitted to the pooled data (y = 0.0004x^2.9608^, R^2^ = 0.9209). (**b**) Log-log plot in which each point represents maximum bite force (standardized here for bite position at jaw tips, see Methods) measured during an experimental session with each specimen represented by a different symbol (*n* = 8). Thin linear regression lines are given for each specimen. The thick dotted regression line was calculated from the pooled data (slope = 3.3269). Estimated bite forces for *Beelzebufo* were calculated using reduced major axis regression (see Methods).

Scaling coefficients of bite force on the linear morphometric variables (log-transformed) ranged from 2.68–4.07, in all cases significantly exceeding the predicted value of 2.0 based on isometric scaling (Table 2). Similarly, the logarithmic scaling coefficient of bite force on body mass (0.91) exceeded that predicted by isometry (i.e., 0.$\mathop{6}\limits^{\bar{} }$). The relationship between bite force and body size (body mass and length), as well as between bite force and head width, did not differ among the individual frogs (ANCOVA with non-significant individual by covariate interactions, Table 3, Fig. 2).

**Table 2:** Results of scaling analysis of bite force on morphometric variables in *Ceratophrys cranwelli*.

Variable	Mean Slope	SE Slope	Lower CL	Upper CL	Intercept Jaw Tips	Intercept Jaw Mid	Isometric Prediction	Allometry Observed
Body Length	2.6835	0.1355	2.5479	2.8190	−4.0231	−3.7220	2.0	+
Head Length	3.3462	0.2176	3.1286	3.5639	−3.7400	−3.4389	2.0	+
Head Width	3.3269	0.1522	3.1747	3.4791	−4.3051	−4.0040	2.0	+
Head Depth	4.0708	0.1986	3.8722	4.2694	−4.0935	−3.7925	2.0	+
Body Mass	0.9105	0.0548	0.8557	0.9653	−0.6455	−0.3445	0.$\bar{6}$	+

**Table 3:** Results of analysis of covariance examining individual variation of bite force performance with respect to each morphometric variable. Non-significant interaction effects (in bold) indicate that bite force does not differ among individual frogs.

Model	F_1, 1, 7_	P
Body Mass	214.34	<0.0001
Individual	2.47	0.0325
Mass × Individual	0.47	**0.8527**
Body Length	321.23	<0.0001
Individual	3.96	0.0021
Body Length × Individual	1.62	**0.1566**
Head Length	222.87	<0.0001
Individual	7.41	<0.0001
Head Length × Individual	3.27	0.0072
Head Width	401.75	<0.0001
Individual	3.45	0.0052
Head Width × Individual	1.89	**0.0962**
Head Depth	311.19	<0.0001
Individual	7.47	<0.0001
Head Depth × Individual	2.85	0.016

Full model and stepwise multiple regressions with bite force as the dependent variable and all of the morphometrics as the independent variables indicate that head width is by far the best predictor of bite force in our sample (full model — F_5, 57_ = 135.56, p < 0.0001, head width: p = 0.0005, all other variables: p ≥ 0.232; forward and backward stepwise models retain only head width and mass — F_2, 57_ = 348.19, p < 0.0001, head width: p < 0.0001, mass: p = 0.0394; partial correlations — head width = 0.457, all other variables ≤ 0.165).

### Estimates of jaw-adductor muscle size in *Ceratophrys* frogs and *Beelzebufo*

**Figure 3:**
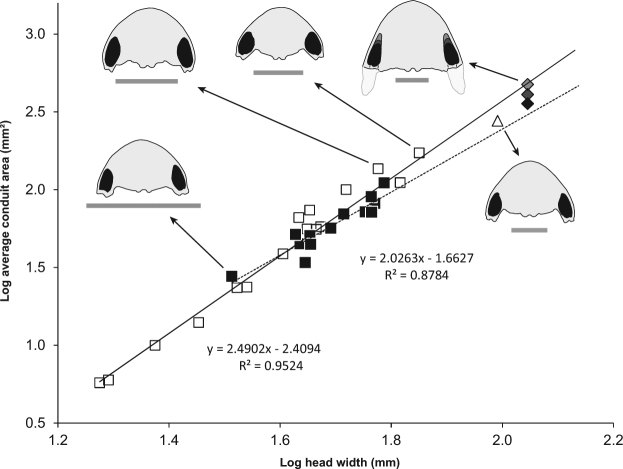
Relationship between head width and size of palatal conduits of major jaw-adductor musculature of ceratophryid frogs. Log-log plot of head width vs. average area of right and left palatal conduits of the major jaw-adductor musculature of *Ceratophrys* spp. based on dissections and skeletal specimens. Solid linear regression line represents all *Ceratophrys* spp. (open squares □, y = 2.4902x − 2.4094, R^2^ = 0.9524). Dotted linear regression line represents only *C. cranwelli* (filled squares ■, y = 2.0263x − 1.6627, R^2^ = 0.8784). Estimate for the exceptional *C. aurita* specimen (LACM 163430) is represented by a triangle (△). Three estimates for the *Beelzebufo* composite reconstruction with a head width of 111 mm (Fig. 3D in ref.^28^) are represented by diamonds (◊), based on conservative, likely, and maximum conduit size. Silhouettes scaled to the same head width, from smallest to largest, are provided for *C. cranwelli* (collection of A.K.L.), *C. ornata* (UMCZ R1529), *C. ornata* (LDUCZ W186), *C. aurita*, and the *Beelzebufo* reconstruction. Scale bars = 40 mm. Note that our estimate of maximum bite force for *Beelzebufo* is based on larger individuals with a head width of 154 mm (estimate of maximum size provided in ref.^28^ based on recovered material).

The ventral aspect of the adductor chambers of frogs is bound by bony elements that form a conduit for the primary jaw-adductor musculature. Because the muscle fibers are near vertically oriented as they pass through the conduit, the area of the pair of conduits in palatal view serves as a gauge of the cross-sectional area of the primary jaw muscles that generate bite force. We found that the average area of the pair of palatal conduits, relative to head width (the external morphometric that best predicts bite force in live specimens), is comparable among *C. cranwelli*, other *Ceratophrys* spp. including the exceptional *C. aurita* specimen, and *Beelzebufo* (Fig. 3; Supplementary Table S2).

### Predictions for other frogs

A pooled RMA regression of log bite force on log head width produced a positively allometric linear scaling coefficient of 3.3269 ± 0.1524. The intercept calculated for a bite position at the jaw tips was −4.2336. At the jaw antero-posterior midpoint (i.e., midpoint between jaw tips and jaw joint measured in parallel to the antero-posterior axis of the skull), approximately the position of the posterior teeth, the intercept was −3.9326. Using regression means for log head width and log bite force at each jaw position, the linear relationship described by the model (log bite force = 3.3269 *log head width – intercept) predicts that *Ceratophrys aurita* specimen LACM 163430 (head width = 98.3 mm) would have had a bite force of 248.6 N at the jaw tips and 497.1 N at the jaw midpoint (Table 4). The same model predicts that a *Beelzebufo* with head width of 111 mm (FMNH PR 2512) would have bite forces at the jaw tips/midpoint of 372.4/744.8 N, and large individuals (head width = 154 mm, UA 9269; ref.^28^) would have bite forces of 1106.8/2213.7 N.

**Table 4:** Estimated bite force of *Ceratophrys aurita* and *Beelzebufo ampinga*. Model generated with pooled reduced major axis (RMA) regression of empirical *in vivo* measurements of bite force on head width in eight *C. cranwelli*.

	Slope	Intercept	Bite Force at Jaw Tips (N)			Intercept	Bite Force at Jaw Midpoint (N)		
			*C. aurita* ^1^	*Beelzebufo* ^2^	*Beelzebufo* ^3^		*C. aurita* ^1^	*Beelzebufo* ^2^	*Beelzebufo* ^3^
Mean	3.3269	−4.2336	**248.6**	**372.4**	**1106.8**	−3.9326	**497.1**	**744.8**	**2213.7**
Lower CL	3.1745	−4.0005	211.3	310.8	878.7	−3.6995	422.6	621.6	1757.5
Upper CL	3.4793	−4.4667	292.4	446.2	1394.1	−4.1656	584.8	892.5	2788.2

^1^LACM 163430, ^2^FMNH PR 2512, ^3^UA 9269. Head width estimates used in calculation of bite force: *Ceratophrys aurita* = 98.3 mm, *Beelzebufo ampinga* = 111 mm and 154 mm. Note that the head width estimate provided here for FMNH PR 2512 (111 mm) differs from that reported in ref.^28^ (129 mm) because the published estimate of head width includes skull roofing bones that extend laterally beyond the jaw joints. SE slope = 0.1524, SE intercept = 0.23308.

## Discussion

Our results demonstrate that extant *Ceratophrys* frogs bite with forces comparable to those of many predatory amniotes (~5 to 500 N at the jaw midpoint). This capacity for forceful biting is associated with prey capture using a large and highly adhesive tongue^8^. Nonetheless, following tongue adhesion large prey must be trapped and restrained with a vice-like clamp of the jaws. In contrast to the gracile skull and flexible mandible typical of most frogs^29^, the skull of ceratophryids is rigid and heavily built, the mandibular symphysis is fused, and the mentomeckelian joints are immovable^30,31^. These characteristics should more effectively transmit jaw-adductor muscle forces to the mandible during a bite, including an enhanced transmission of muscle forces from the balancing to the working side of the jaws during a unilateral bite. Furthermore, extant ceratophryids have extremely short jaws (i.e., short jaw out-lever) thus providing great mechanical advantage for biting. Greater bite forces permit access to a broader range of prey in predators that use their jaws to subdue prey^32,33^.

**Figure 4:**
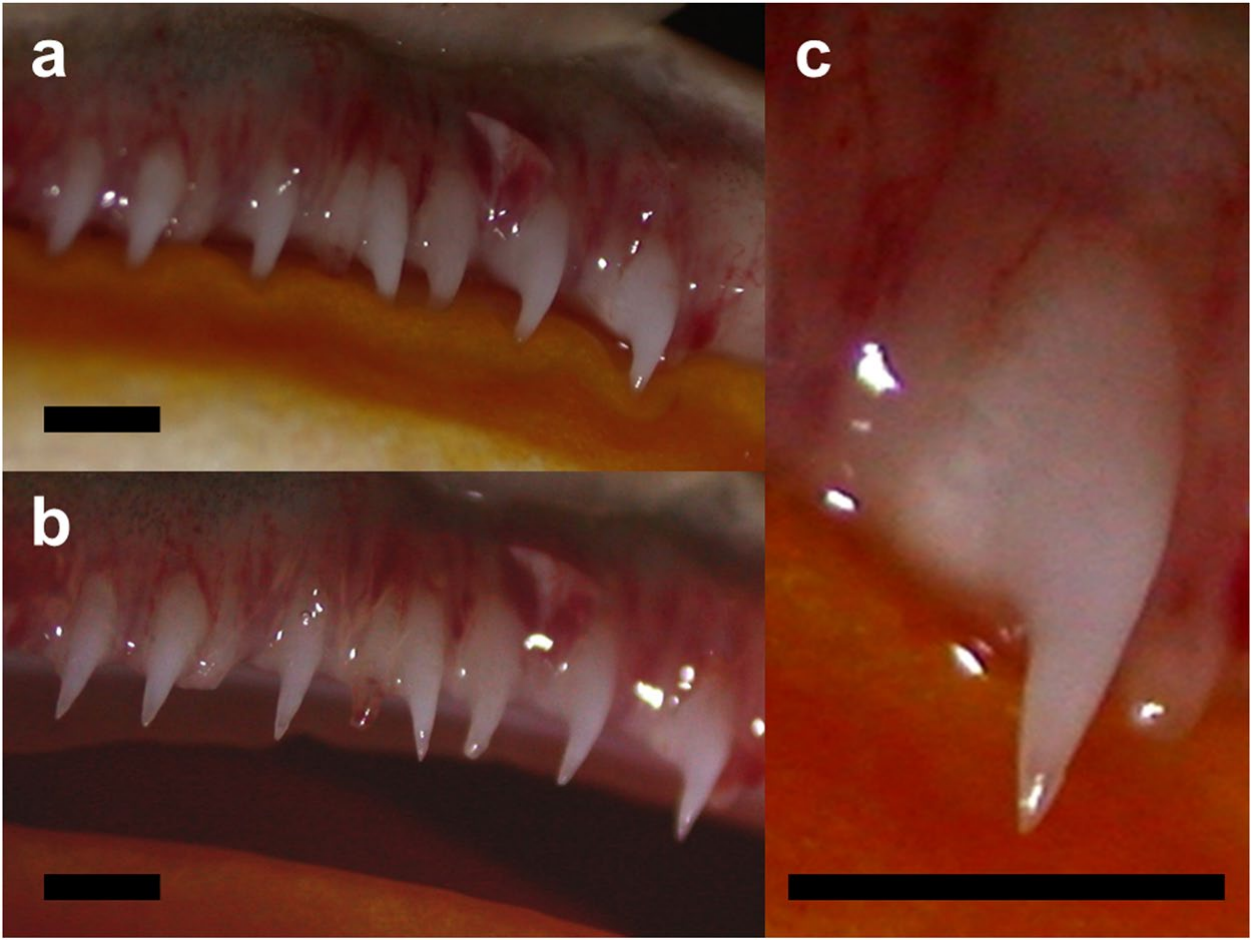
Teeth of *Ceratophrys cranwelli*. As with almost all other extant frogs bearing teeth, *Ceratophrys* has teeth only on the upper jaw. Unusual among frogs, the teeth of *Ceratophrys* exhibit a derived non-pedicellate morphology and have sharp recurved tips situated upon robust, labiolingually expanded bases. (**a**) view of teeth with jaws closed; (**b**) view of teeth with mouth slightly open; (**c**) close-up of single tooth. Scale bars = 1 mm.

Complementing the musculoskeletal robustness of the jaws, the unicuspid and non-pedicellate teeth of *Ceratophrys* ^31,34,35^ are unusual for frogs, and exhibit structural characteristics that reflect their function during prey dispatch (Fig. 4). Having sharp tips and being strongly attached to the premaxillae and maxillae^27,31^, they are well-suited to produce high pressures to effectively penetrate and engage the integument of prey. They also are recurved, labiolingually expanded, and have robust bases, and thus are ideally shaped to resist forces produced by struggling prey and prevent it from escaping. In addition, *Ceratophrys* has a pair of large and fully ossified odontoids (fangs) on either side of the mandibular symphysis, which enhance the ability to secure large, strong prey^28,31^. In contrast to theoretical predictions, we found that in *C. cranwelli* bite-force performance scales with strong positive allometry with respect to both body size and head dimensions. Given that the physiological cross-sectional area of a muscle is a major determinant of its capacity to generate force^36^, and assuming isometric growth and maximum voluntary performance throughout ontogeny, it follows that bite force should scale isometrically with the sum of the cross-sectional areas of all of the jaw-adductor muscles contributing to a bite. Because areas scale to the square of linear measurements, bite force also is predicted to scale to the square of linear measurements (slope = 2.0)^14^. Moreover, as areas scale to the two-thirds power of volumes, and volume scales isometrically with mass (approximately for most live vertebrates)^36^, bite force is predicted to scale to the two-thirds power of body mass (slope = 0.$\mathop{6}\limits^{\bar{} }$)^14^. In *C. cranwelli*, the scaling exponents between bite force and linear morphometrics of body and head size, as well as between bite force and body mass, are significantly greater than theoretical predictions.

The positive allometric relationship in *Ceratophrys* between bite force and body and head size is consistent with results found for other taxa examined to date. For example, positive intraspecific allometry between *in vivo* voluntary bite force and body and head morphometrics is observed in fish^22^, lizards^13,17^, tuatara^37^, alligators^14^, turtles^17,21^, and rodents^38,39^ (Table 1). Published intraspecific scaling analyses of voluntary bite force in mammals other than rodents are rare, but see analyses of scaling of bite force with age in hyenas (including scaling coefficients)^40^ and in coyotes^41^.

Our predictions of bite-force performance at two different jaw positions for *C. aurita* specimen LACM 163430 (~250–500 N) and *Beelzebufo* (~370–740 N [FMNH PR 2512, HW = 111 mm] and ~1100–2200 N [UA 9269, HW = 154 mm]) are consistent with previously published studies of bite force in other medium to large-sized vertebrates. Direct empirical *in vivo* measurements of voluntary bite force for vertebrates with heads of comparable size to that of *C. aurita* (LACM 163430) and *Beelzebufo* are uncommon. However, useful comparisons are afforded by several *in vivo* scaling analyses on turtles and crocodylians, as well as measurements and estimates of bite force for carnivorous mammals. A specimen of the large predatory turtle *Chelydra serpentina* (common snapping turtle), with a mass of 16.65 kg and a head width of 101 mm, was measured to bite with a force of 657 N^17^. This exceeds the estimated maximum bite force of *C. aurita* (LACM 163430) with a head of slight lesser width (98.3 mm, 497.1 N) by ~32%. Although the position in the jaw at which bite forces were measured was not reported (i.e., bite out-lever^10^), the scaling relationship provided by the authors predicts that a *C. serpentina* with a head 154 mm wide (~73.5 kg body mass) should bite with a force of 2042 N, similar to that of a *Beelzebufo* with a head of equal width (2213.7 N).

For a given head width, maximum bite-force performance in crocodylians is greater than calculated for *C. aurita* but less than calculated for *Beelzebufo*. For crocodylians with a head width of 98.3 mm, corresponding to *C. aurita* (LACM 163430), bite forces estimated at the position of the largest tooth at the posterior end of the tooth row are 689.3 N for *Alligator mississippiensis* (American alligator, interpolated from empirical data of bite force at 11^th^ maxillary tooth; ref.^14^), 680.5 N for *Crocodylus porosus* (saltwater crocodile, interpolated), and 707.7 N for *C. johnstoni* (freshwater crocodile, extrapolated) (G. M. Erickson and AKL unpublished data, see ref.^24^ for relationships between bite force and body size). These crocodylians would have masses of 8.2 kg, 11.9 kg, and 9.4 kg, respectively. In all cases, our interpolation/extrapolation of bite force relative to head width for crocodylians exceed our bite force estimate for *C. aurita* (LACM 163430) by approximately 37–42%. For crocodylians with a head width of 154 mm, corresponding to that of the largest specimen of *Beelzebufo*, bite force estimates are 1659.6 N for *Alligator mississippiensis*, 1836.5 N for *Crocodylus porosus*, and 1863.6 N for *C. johnstoni* (G. M. Erickson and AKL unpublished data, see ref.^24^). These crocodylians would have masses of 24.2 kg, 34.0 kg, and 27.3 kg, respectively. In contrast to the comparison of bite force between crocodylians and *C. aurita* (LACM 163430), our estimate for the maximum bite force of *Beelzebufo* exceeds the bite force of crocodylians with a comparable head width by approximately 16–25%.

Our predictions for *Beelzebufo* also are compatible with available information on bite force in mammals. Published direct empirical measurements of voluntary bite forces in large mammals are surprisingly rare. Studies on captive colonies of two carnivorans, *Crocuta crocuta* (spotted hyaena)^40^ and *Canis latrans* (coyote)^41^, provide the only reliable empirical data. *C. crocuta* produced values of ~1000–4500 N for 6 + year old adults^40^. The greatest measurement for *C. latrans*, produced by an eight year-old male, was 704 N^41^. Bite forces estimated for *Beelzebufo* (UA 9269), both at the jaw tips and jaw midpoint, span the lower half of the range for *C. crocuta* and considerably exceed the maximum for *C. latrans*. Estimates of bite force derived from morphology-based models for *Canis lupus* (gray wolf), and *Panthera leo* (lion) and *P. tigris* (tiger) the size of adult females, are 774 N, 2024 N, and 2165 N, respectively^32^. Our estimates of bite force at the jaw tips for *Beelzebufo* (UA 9269) exceed the estimated bite force for *C. lupus*, the latter of which is unexpectedly similar to the *in vivo* measurement for the much smaller *C. latrans*. Bite force at the jaw midpoint for *Beelzebufo* (UA 9269) is similar to that estimated for the largest extant cats, *P. leo* and *P. tigris*.

Extant ceratophryid frogs are exceptional in having a disproportionately large head^30^, reflecting their megalophagous, ambush predatory tactics. They also have extremely wide skulls^29^, relative to skull length and depth^30^, which house large jaw adductor muscles. Although ceratophryids generally possess these characteristics, research on interspecific variation of the jaw-adductor mechanics of these frogs is warranted, including among species of *Ceratophrys*, which appear to exhibit appreciable interspecific variation of skull proportions.

Evaluating the biology of long-extinct animals, such as making estimates of their performance capacities, is challenging due to the incomplete nature of fossil material, as well as the desire to identify appropriate modern analogues. Although *Beelzebufo* is strikingly similar to *Ceratophrys* in many ways (e.g., unicuspid teeth, robust skull, cranial exostosis, absence of a palatine shelf on the premaxilla), available material suggests that its skull was relatively longer and shallower, which might also indicate differences in jaw muscle architecture^28^. Therefore, our estimates of its bite-force performance should be received with caution, as should be the case with any such estimate. Nevertheless, the head width we use for *Beelzebufo* (154 mm) may be less than the maximum possible as some fossil material corresponding to frogs of this size have unfused cranial sutures in contrast to the strongly fused cranial bones of adult *Ceratophrys* ^27^. Given that prey size is known to increase with body size in a variety of tetrapods^42^, and that *Beelzebufo* clearly had the ability to bite with considerable force, large individuals would have been able to prey upon a variety of contemporaneous taxa, including small/juvenile crocodiles and non-avian dinosaurs^43^.

## Methods

### Morphometrics

Body mass (BM) and body length (BL) were measured and recorded as indicators of body size. Head size was quantified by measuring three variables: head length from the jaw joint to the tip of the snout (HL), head width at the lateral extent of the jaw joints (HW), and head depth (HD) from the dorsal-most part of the skull just posterior of the orbits to the ventral extent of the lower jaw. All head measurements were made parallel or perpendicular to the antero-posterior or dorsal-ventral axis of the head to avoid the potentially confounding effects of angular measurements^44^. Linear measurements were made with digital calipers to the nearest 0.1 mm, and body mass was measured to the nearest 0.1 g with a digital scale.

### Specimens and data sampling for *in vivo* bite-force experiments

Data were collected from eight individuals of the ceratophryid frog *Ceratophrys cranwelli* during post-metamorphic ontogeny. Specimens were purchased from commercial dealers and donated by private breeders. Data sampling followed a longitudinal experimental design. Over a period of one year of post-metamorphic growth, we conducted a series of experimental sessions (5–10 per animal, mean = 7.1) during which morphometric and bite-force data were collected. The pugnacious nature of *Ceratophrys* and its willingness to bite as a defensive response^29,45^, unusual among amphibians, makes this taxon an excellent subject for an analysis of bite force (Fig. 1). All procedures in this study were approved by the Animal Care and Use Committee of California State Polytechnic University, and the methods were carried out in accordance with the relevant guidelines and regulations.

### Bite-force performance

Bite-force performance was measured using a piezoelectric force transducer (type 9203, Kistler, Switzerland) custom fitted with stainless steel bite plates and connected to a charge amplifier (type 5995, Kistler, Switzerland)^11^, but see refs^10,46^. The transducer was prepared for experiments by adhering leather strips at the ends of the plates where the frogs were induced to bite^10^ (Fig. 1). The leather served to protect the animals’ teeth and jaw bones from damage, provide a surface for the frog to grip with its jaws, and avoid the potential for reduced performance via sensory feedback if a non-naturalistic surface (e.g., steel) was used^10^. To calibrate amplifier output, a series of weights suspended with fishing line on the leather strip was used to reflect the force applied during bites to the same area. The distance between the plates and the thickness of the leather strips was adjusted as needed so that gape angle was approximately consistent among individuals (20–25 degrees). Three bite-force trials were performed on each frog during each experimental session, with one minute of rest between each trial. All frogs bit the transducer vigorously during all trials.

We standardized for variation, both among trials and among individuals, in the position along the jaw line that engaged the leather strips on the bite plates by using a simple lever calculation to compute bite force at two bite points^10^, at the tips of the jaws and at the antero-posterior midpoint of the lower jaw (i.e., midpoint between jaw tips and jaw joint), with the latter approximately corresponding to the location of the posterior teeth. These two bite points encompass the possible range of bite forces during a high-effort predatory episode.

### Statistical analysis

All data were log-transformed and found to be normally distributed (Shapiro-Wilk W) for subsequent parametric statistical analyses^47^. Analyses were performed using JMP v8.0.2 and SAS v9.3.

Because our study was based on a longitudinal experimental design, we tested for individual variation in the relationship between bite force and each morphometric. To do this we used ANCOVA with bite force as the dependent variable, individual as the factor, morphometric as the covariate (separate model for each morphometric), and an individual by morphometric interaction. When the individual by morphometric (covariate) interaction was not significant, indicating that the slope of the relationship between the morphometric and bite force did not differ among the individual frogs, it was removed from the model. For these cases, the pooled data across all specimens is thus representative of the ontogenetic pattern for the individual frogs.

### Tests of allometric scaling

To test the hypothesis that *Ceratophrys* exhibits a positive allometric increase in bite-force during ontogeny with respect to the measured morphological variables, we performed a reduced major axis (RMA) regression of log bite force on the log of each morphometric variable for each individual frog. We then pooled the slopes to produce a linear scaling coefficient for each bite force versus morphometric relationship. The standard error of the pooled RMA slope for each relationship was estimated by bootstrapping with 10000 iterations (i.e., 10000 bootstrap samples generated for each individual pooled to calculate 10000 slopes). Intercepts were calculated for bite positions at the jaw tips and at the jaw midpoint using regression means for the log of bite force on the morphometric at each bite position.

### Estimates of jaw-adductor muscle size in *Ceratophrys* frogs and *Beelzebufo*

We examined the cranial musculoskeletal anatomy of *Ceratophrys* frogs to identify factors potentially significant in determining bite-force performance that also could be ascertained in skeletal or fossil material. The size of the adductor chamber of frogs is likely to have a strong association with potential bite-force performance. At the ventral end of the chamber on each side of the skull, bony elements form a conduit for the passage of the primary jaw-adductor musculature. This conduit can therefore be used as a proxy for the cross-sectional area of the adductor musculature (Fig. 3). The major jaw adductor muscles originate from the braincase and adjacent bones. From their origins, the muscle fibers run towards the palatal conduits, assume vertical or near vertical orientations via a pulley arrangement, and insert onto the dorsomedial and lateral aspects of the posterior end of the mandible. This pair of conduits through which the fibers of the major jaw-adductor muscles pass on their way to inserting onto the mandible provides an estimate of the cross-sectional area of the muscles that generate bite force, one that can be acquired from skeletal material alone and thus applied to comparable forms (i.e., *C. aurita* and *Beelzebufo*). We quantified and compared the area of the pair of palatal conduits among *C. cranwelli*, other *Ceratophrys* spp. including the exceptional *C. aurita* specimen, and *Beelzebufo* (Supplementary Figure S3), to assess relative similarity in the cross-sectional area of the major jaw-adductor musculature contributing to bite-force performance. Due to the incomplete nature of the existing *Beelzebufo* material, we made three estimates of palatal conduit area (conservative, likely, maximum; Fig. 3).

### Extrapolation of bite forces for exceptional *Ceratophrys* and extinct *Beelzebufo*

Using the results of our scaling analysis, we extrapolated bite force for two larger frogs. First, we estimated the maximum bite force of an exceptionally large *Ceratophrys* specimen housed at the Natural History Museum of Los Angeles County (LACM 163430) (see ref.^28^; Supplementary Figure S4). This is a no data skeletal specimen indicated as *C. varia*, which is an invalid junior synonym for *C. aurita* (Brazilian Horned Frog)^48,49^. *C. aurita* is considered the largest species in the genus^50^, and we found that LACM 163430 has a body length of 178 mm (estimated by the length of the skull plus the articulated vertebral column and pelvic girdle) and a head width of 98.3 mm. We also make a prediction for the bite force of the extinct *Beelzebufo ampinga* from the Late Cretaceous of Madagascar. This is a large anuran (body length = 232 + mm, skull width = 111–154 + mm; ref.^28^; Supplementary Figure S5), which extensive phylogenetic analyses recovered as more closely related to ceratophryids than any other extant lineage^27,28^.

To estimate the bite force of LACM 163430 and of *Beelzebufo*, we determined the best morphometric predictor of bite force (i.e., head width – see Results) by running full model and stepwise multiple regressions with bite force as the dependent variable and all of the morphometrics as the independent variables. We then performed a reduced major axis (RMA) regression of log bite force on log head width, calculated the standard error of the pooled RMA, and used a bootstrapping method to calculate the intercepts for bite positions at the jaw tips and at the posterior extent of the tooth row, as described above. The linear relationship described by the model was used to calculate the potential bite-force performance of a *Ceratophrys* with a head width of 98.3 mm (LACM 163430), as well as to make two estimates of the bite force of *Beelzebufo*, one with a head width of 111 mm (FMNH PR 2512) and one with a head width of 154 mm (UA 9269; see ref.^28^).

### Data availability

The data that support the findings of this study are available from the authors on reasonable request.

## Electronic supplementary material

Supplementary Information
