# Supplementary material for: Bite force in the horned frog (Ceratophrys cranwelli) with implications for extinct giant frogs
Source: Sci Rep. 2017 Sep 20;7:11963. doi: 10.1038/s41598-017-11968-6 (PMC5607344; doi:10.1038/s41598-017-11968-6)
Supplement: Supplementary file 1 — Supplementary Information [file 41598_2017_11968_MOESM1_ESM.pdf]

Supplementary Information

Bite force in the horned frog (*Ceratophrys cranwelli*) with implications for extinct giant frogs

A. Kristopher Lappin[1*], Sean C. Wilcox[1,2], David J. Moriarty[1], Stephanie A. R. Stoeppler[1†], Susan E. Evans[3], and Marc E. H. Jones[4,5]

[1] *Biological Sciences Department, California State Polytechnic University, Pomona, CA 91768, USA*

[2] *Department of Biology, University of California, Riverside, CA 92521, USA*

[3] *Department of Cell and Developmental Biology, University College London, London, United Kingdom*

[4] *School of Earth and Environmental Sciences, The University of Adelaide, South Australia, 5005, Australia*

[5] *South Australian Museum, Adelaide, South Australia, 5000, Australia*

[†]*in memoriam*

[*]*Correspondence to aklappin@cpp.edu*

Supplementary Table S1. External morphometrics and measurements of bite force for scaling analysis of *Ceratophrys* cranwelli.

Specimen	BodyLength (mm)	HeadWidth (mm)	HeadLength (mm)	HeadDepth (mm)	BodyMass (g)	BiteForce_JawMidpt (N)	BiteForce_JawTips (N)
4	52.2	27.6	21.2	14.5	22.5	6.6	3.3
4	55.6	31.2	22.6	15.7	31.1	9.2	4.6
4	61.2	32.9	24.9	16.0	32.3	11.3	5.6
4	67.5	35.1	26.1	17.1	39.9	14.0	7.0
4	68.8	36.2	26.4	17.3	56.2	15.8	7.9
4	74.5	38.3	27.0	18.2	63.3	19.9	9.9
4	75.0	38.7	27.0	18.3	80.3	24.0	12.0
4	78.4	40.8	29.3	19.2	96.6	22.2	11.1
5	58.6	33.0	23.2	16.4	30.6	9.0	4.5
5	57.9	33.2	23.8	16.6	31.0	10.0	5.0
5	62.9	35.0	24.7	16.9	36.6	12.9	6.5
5	63.8	36.9	24.9	17.1	43.7	14.8	7.4
5	63.8	37.3	25.1	17.5	40.3	16.1	8.0
5	80.1	42.7	28.5	19.7	82.2	24.6	12.3
5	83.5	44.5	30.5	19.8	95.5	26.9	13.4
5	85.3	44.9	31.1	20.6	131.0	32.9	16.4
6	44.5	24.0	16.9	11.7	13.7	3.4	1.7
6	48.2	26.6	17.7	13.2	21.8	4.2	2.1
6	49.9	27.1	17.7	13.6	22.7	6.1	3.0
6	54.9	29.6	20.2	14.2	21.9	7.9	3.9
6	57.7	30.2	20.3	14.6	23.0	9.6	4.8
6	61.4	32.8	21.0	15.4	32.8	10.9	5.4
6	61.4	33.2	21.8	15.9	36.0	14.2	7.1
6	74.6	37.6	24.9	17.1	57.2	15.7	7.8
6	76.6	39.0	26.4	18.0	59.1	18.1	9.1
6	95.6	46.1	31.7	20.5	147.8	30.1	15.0
7	46.5	28.5	19.3	14.2	22.2	8.6	4.3
7	49.7	28.8	19.4	14.4	22.0	8.3	4.2
7	54.1	32.4	19.6	14.9	29.1	10.5	5.3
7	55.8	33.0	19.9	15.3	36.6	11.5	5.8
7	70.1	38.0	22.7	16.8	56.6	16.4	8.2
8	52.0	30.8	18.3	14.0	22.2	9.9	4.9
8	57.6	32.3	20.3	14.6	28.0	13.0	6.5
8	61.8	32.9	21.0	14.6	34.6	13.8	6.9
8	68.1	36.3	24.1	16.5	44.0	17.1	8.5
8	71.4	38.7	24.9	16.7	64.9	20.3	10.2

Supplementary Table S1 (continued).

Specimen	BodyLength (mm)	HeadWidth (mm)	HeadLength (mm)	HeadDepth (mm)	BodyMass (g)	BiteForce_JawMidpt (N)	BiteForce_JawTips (N)
9	49.1	31.4	19.7	13.9	23.1	8.0	4.0
9	51.1	31.6	19.9	14.2	23.6	7.6	3.8
9	55.7	33.3	21.3	14.8	30.9	11.9	5.9
9	60.7	33.7	21.5	15.1	34.8	12.0	6.0
9	65.8	36.9	23.2	16.0	40.8	12.3	6.1
9	66.5	38.8	23.7	17.4	56.8	15.5	7.7
9	71.3	41.0	25.4	17.4	61.3	21.1	10.5
10	52.8	29.4	18.2	13.7	22.0	8.7	4.3
10	54.2	31.7	20.3	14.7	24.6	9.7	4.9
10	56.0	32.0	20.3	15.0	26.0	10.2	5.1
10	68.7	35.5	21.7	15.7	37.2	13.6	6.8
10	69.0	36.5	22.2	16.2	39.7	17.8	8.9
10	76.9	41.0	26.1	18.6	50.8	17.7	8.8
10	77.7	42.5	28.4	19.1	74.2	21.2	10.6
10	84.2	43.8	28.4	19.1	80.3	23.4	11.7
10	86.0	45.6	31.0	20.4	91.7	25.8	12.9
13	39.8	22.4	14.7	10.8	8.9	2.7	1.4
13	45.7	24.9	17.0	11.9	14.9	6.6	3.3
13	47.9	26.1	17.8	12.5	15.2	7.4	3.7
13	56.8	28.4	19.1	13.7	29.8	7.3	3.6
13	56.9	28.6	19.1	13.7	25.8	9.0	4.5
13	59.7	29.9	21.0	13.9	28.1	8.5	4.2

Supplementary Table S2. Head width and conduit areas for morphometric analysis of *Ceratophrys* and *Beelzebufo.*

Species	SI Specimen	HeadWidth (mm)	Conduit_Right (mm^2)	Conduit_Left (mm^2)	Conduit_Average (mm^2)
C. calcarata	LACM_163429	43.0	63.7	68.7	66.2
C. cornuta	A08	18.8	5.6	5.9	5.7
C. cornuta	A09	19.6	5.9	6.1	6.0
C. cornuta	B01	28.4	14.9	13.1	14.0
C. cornuta	B02	23.7	10.3	9.6	10.0
C. cornuta	B03	33.3	23.0	24.0	23.5
C. cranwelli	A03	58.9	79.9	83.5	81.7
C. cranwelli	A04	43.3	43.7	46.1	44.9
C. cranwelli	A05	43.2	44.7	45.0	44.9
C. cranwelli	A06	56.7	66.5	77.3	71.9
C. cranwelli	A07	45.1	47.2	54.4	50.8
C. cranwelli	A10	44.2	35.4	32.7	34.0
C. cranwelli	A12	58.2	63.2	80.3	71.7
C. cranwelli	A13	42.4	53.2	49.9	51.5
C. cranwelli	A14	49.1	51.7	61.4	56.5
C. cranwelli	A15	45.2	46.4	42.7	44.5
C. cranwelli	A16	51.8	66.6	73.0	69.8
C. cranwelli	A17	61.3	105.3	116.1	110.7
C. cranwelli	B04	58.1	93.0	87.6	90.3
C. cranwelli	B05	32.6	27.6	28.0	27.8
C. ornata	B06	34.7	20.8	26.4	23.6
C. ornata	B07	42.2	49.6	52.4	51.0
C. ornata	B08	46.3	53.6	57.6	55.6
C. ornata	B09	47.1	60.4	55.3	57.9
C. ornata	LACM_163427	45.0	75.7	72.2	73.9
C. ornata	AKL_2099	65.5	106.5	115.5	111.0
C. ornata	UMCZ_R1529	59.8	131.1	141.7	136.4
C. ornata x cranwelli	A01	40.3	38.3	39.1	38.7
C. ornata x cranwelli	A11	44.5	56.4	55.4	55.9
C. spp	LDUCZ_W186	70.9	169.4	175.7	172.5
C. spp	UMCZ_R1530	52.3	96.2	103.9	100.1
C. aurita	LACM_163430	98.3	272.6	282.6	277.6
B. ampinga	Evans et al. 2015: Fig 3D	111.0	349.0	365.4	357.2
B. ampinga	Evans et al. 2015: Fig 3D	111.0	396.0	422.6	409.3
B. ampinga	Evans et al. 2015: Fig 3D	111.0	468.2	481.2	474.7

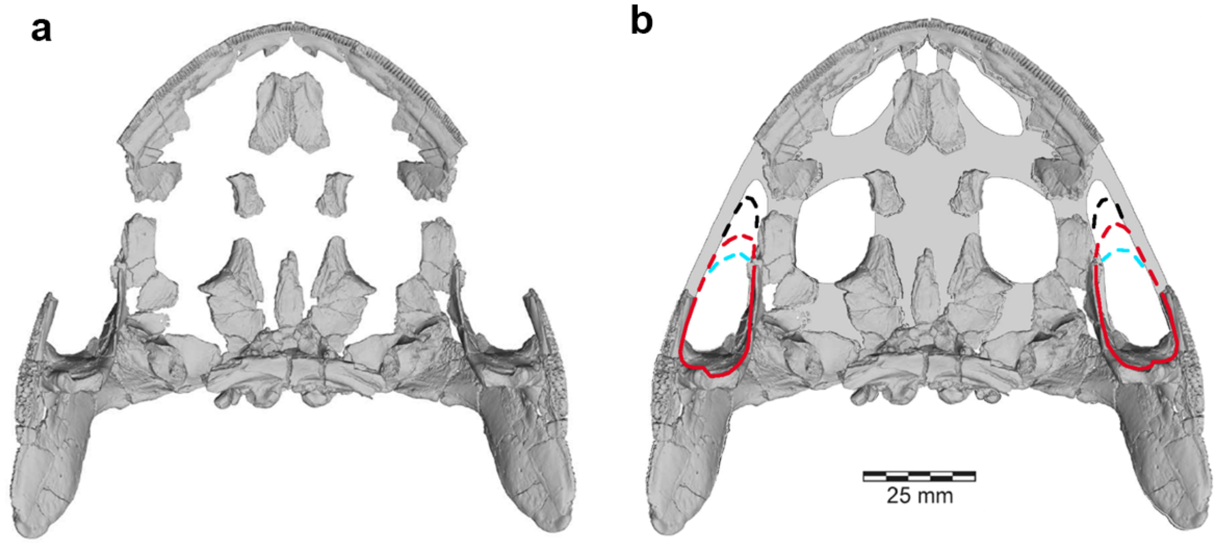

Supplementary Figure S3. Reconstructions of *Beelzebufo* (FMNH PR 2512) modified from Evans et al. (2014). (a) ventral view, (b) ventral view showing estimates of adductor conduit area (conservative, likely, maximum).

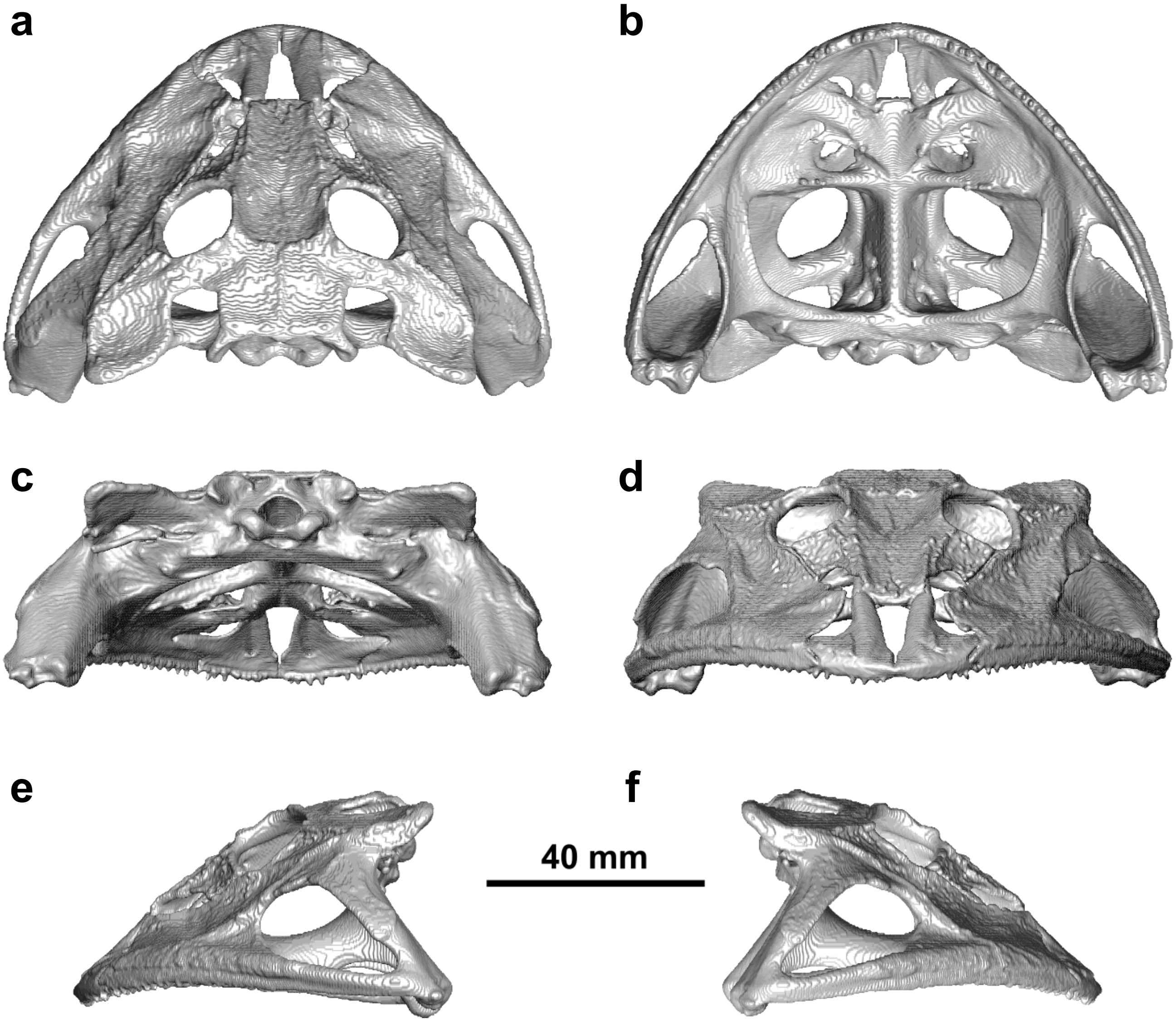

Supplementary Figure S4. CT scan of *Ceratophrys aurita* specimen LACM 163430. (a) dorsal, (b) ventral, (c) posterior, (d) anterior, (e) left lateral, and (f) right lateral view.

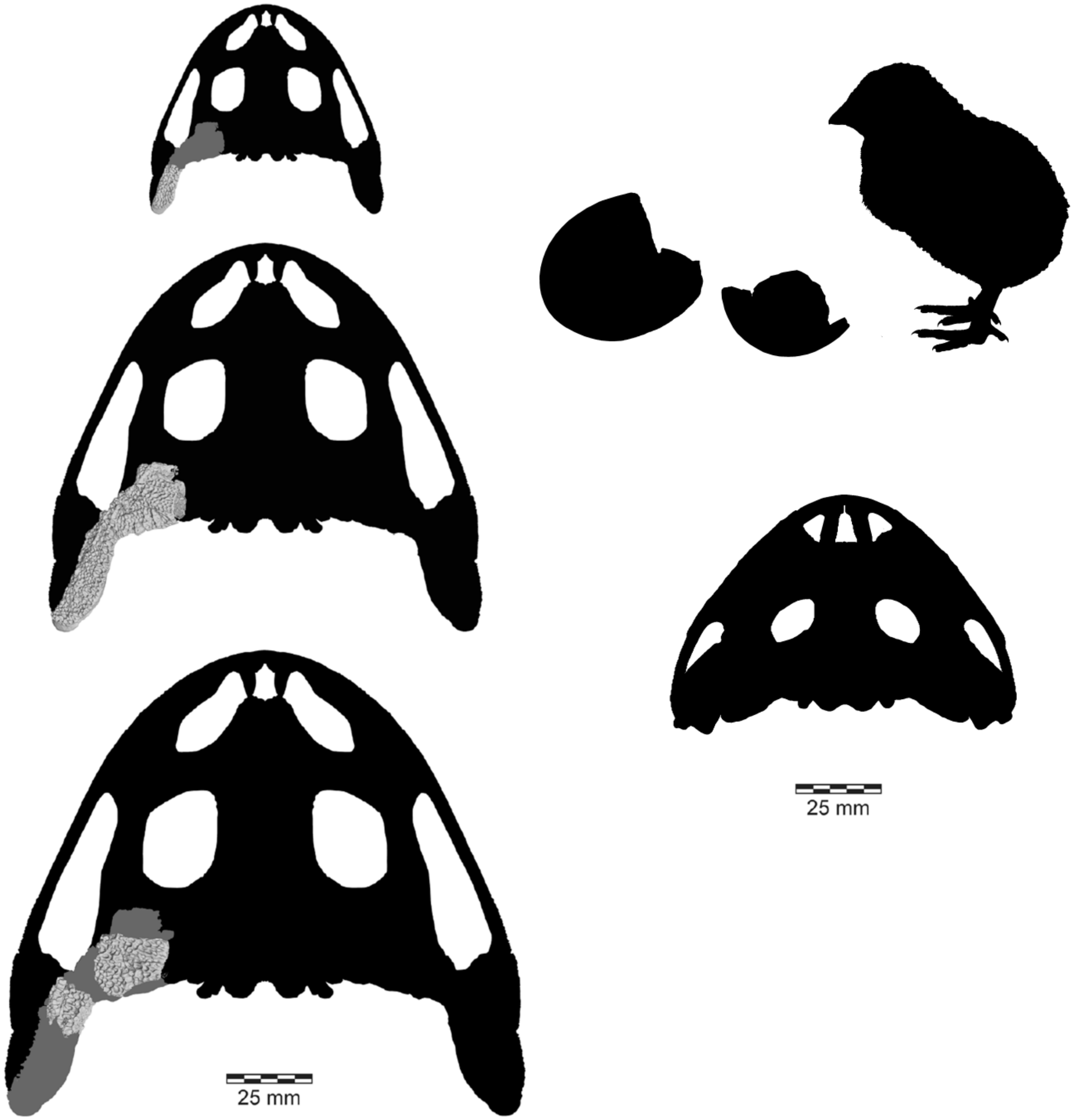

Supplementary Figure S5. Size comparison of three reconstructions of extinct *Beelzebufo ampinga* from Fig. 47 in ref. 28 (left) and exceptional extant *Ceratophrys aurita* LACM 163430 (right) with newly hatched domestic chicken and egg shell.
